# American Dental Association

**Published:** 1897-01

**Authors:** 


### Reports of Society Meetings.

### AMERICAN DENTAL ASSOCIATION.

(Continued from page 806.)

*August 5, 1896.—Second Day.—Evening Session.*

Meeting called to order at 8.15 by the President, Dr. Crawford.

The minutes of the previous session were read and approved.

Dr. C. N. Peirce, on behalf of the Executive Committee, reported as follows:

The communication from the Publication Committee was read and duly considered, and upon unanimous recommendation, the transactions are again placed in the hands of the S. S. White Manufacturing Company for publication, with the distinct understanding that they shall be issued to the members of the Association at a date not later than February 1, 1897.

Regarding the one hundred dollars asked for by the Committee on National Museum and Library, it was, upon motion, recommended that a sum not exceeding this amount be appropriated, and the treasurer be authorized to pay on presentation of attested voucher for the same. The bill from the widow of J. J. R. Patrick was carefully considered. The committee recommend that in view of the fact that three hundred dollars had been paid Dr. Patrick, and also several sums from other sources for which no credit was given in the account, that the same be not paid, and that a communication be sent to Mrs. Patrick stating that the bill forwarded by her to the Association was referred to the Executive Committee with full power, and that the said committee having a full appreciation of the valuable services rendered by Dr. Patrick, not only to this Association, but to the profession at large, regret their inability to recognize the amount of the claim. They are influenced in this judgment by the fact that the duty to the trust imposed upon them necessitate that they shall treat this matter in a truly business aspect only. The account has been settled in full, Dr. Patrick having drawn from the Association all moneys expended on their behalf and in their interest. The committee therefore consider that the claim cannot be recognized.

The bill from the S. S. White Manufacturing Company for $95.58 is to be paid on the endorsement of the Auditing Committee.

In view of the increased duties of the secretary, and his long and efficient service, it is recommended that the salary for the present incumbent for the coming year be three hundred dollars to enable him to procure such assistants as he may deem desirable.

The Executive Committee recommend that within twenty days after the transactions have been received by the members of the Association, the officers elected in the several sections shall notify the recording secretary of their acceptance of the position, and of the names of the members of their sections, and that sixty days thereafter the president or secretary of the section shall send to the recording secretary of the Association, as far as possible, the titles of papers and the prospective work they have to offer at the stated sessions of this Association.

Report adopted.

Dr. Grant Molyneaux, on behalf of the committee appointed to investigate the claims as to the priority of invention of an electric oven for fusing porcelain, reported that the first practical and public demonstration of an electric oven for fusing porcelain crowns and continuous-gum dentures occurred in October, 1894, Dr. L. E. Custer using an oven of his own invention and construction at the office of Dr. Haskell. Dr. Custer also used an oven of his invention in 1889. The committee believe that Dr. Custer was the first one to practically fuse porcelain in an electric oven, and recommend that this Association accord him that honor.

*Dr. Crouse*, of Chicago.—A year ago I gave the history of the litigation of the Dental Protective Association up to that date. At that time we had our testimony in and were waiting to have the case tried on the low bridge patent. As you know, the Crown Company had had a decision in their favor on the low bridge patent before, and we had been unable to get the case in any other court except the one in which they had obtained their decision. It was finally to be heard by Judge Wheeler, of Brattleboro', Vermont, in February. The case was four days on trial. We went into it with the greatest amount of doubt, from the fact of the decision having been rendered in favor of the patent before the

Superior Court of that Federal district. I am informed that there are only two or three cases on record where a judge occupying a lower position comes into the higher courts and reverses the decision of the higher courts, and ours happens to be one of the cases. When I left the court-room after Mr. Offield, our attorney, had made his argument I felt sure we would win our case, and the other side felt sure they would win. We arranged to have the clerk telegraph us as soon as a decision was rendered. Late in March we received a telegram stating that we had won our case. The next morning one of the attorneys for the Crown Company was in Mr. Offield's office to take testimony. They had not heard that the decision was rendered against them, and when he saw this telegram he left the office and went back to New York. This placed us on an even footing with the Crown Company in the next Federal court. Then their next effort was to get it on appeal, and the next month they brought us to New York on the plea that they had been deprived of their royalty for eight or nine years by this Dental Protective Association, and that the patent only had about two years to run before the time expired, and on that ground they wanted their case heard at once. We filed a counter affidavit to the effect that we had been trying to get them to try their case in some other court. I also filed another affidavit which may not seem important to you, but it is so, nevertheless. It was to the effect that I had knowledge sufficient to believe that there were parties back of this litigation outside of the Crown Company. In the answer the attorneys made no reference whatever to my affidavit, as they did not dare to deny it. The court overruled their motion to put the case on the calendar at once, and we went home, and after we were home about a week we got word again to come back. We sent a long telegram to the court stating it was impossible for us to get there on Tuesday when the case was to be heard, and that we could not come before Wednesday; and we also sent a telegram that, according to Judge Wallace's statement, we supposed that Judges Wallace and Shipman were unable to try this case. We went to New York, and the case was called Wednesday morning. We expected to try it, but we found that Judges Wallace and Shipman had decided not to sit in the case, but that Judges Lacrone and Townsend, of New Haven, would try it. We decided to go on with it. Mr. Dickerson, who had not appeared before, came in and said he represented other parties, and referred to the decision made before. The judge passed upon the points that had not been passed on before. He came down to the point that the low bridge was not patentable, and that the cases of prior use that we had proved showed that the invention was not new. The case has been argued, and we have not received our decision yet. We expected to have it before this meeting, but we have no doubt about how it will be. We expect a favorable decision. Suppose we did not? It would not be wise for me to state what our course would be. Suppose we do get a favorable decision? That makes it impossible for the Crown Company to bring any further suits against members of the profession in the New England States; but if they wish to bring suits against other members of the profession in other parts of the country they can do so, and we will have to fight it further. The object of the Dental Protective Association is to band the profession together, so they can take care of themselves. Some three or four years ago the companies stated that they were tired of litigating, and were willing to take a sum of money and let us alone. The manufactured articles, under cover of patents, that the dental men were paying big prices for, were a greater abuse than the royalty demanded for using certain things; and, with a view of remedying that and banding the profession together, this Association was organized. I feel sure that the time will come when the members of the Association will see the importance of this movement and help along. If each member of the Association will take one share of stock, it is all I ask. The royalty that the companies will get from members of the Dental Protective Association is at an end. One office in New York, a firm of two, has paid the Crown Company over six thousand dollars in royalty since the Association was organized. They have paid that money according to their sworn statement as witnesses on the other side. The Dental Protective Association has recently been sued by the patentees and owners of the Donaldson canal-cleanser. We have prepared an instrument, and we are making it as good as any other, and furnishing it to the profession at one dollar and fifty cents per dozen. I would like to have another meeting of the members of the Dental Protective Association at another time, and we will try to arrange it for some time when it will not interfere with the regular sessions of the Association.

*Dr. Brophy.*—I wish to speak about the paper of Dr. Fillebrown. It is very interesting, and will excite a great deal of comment no doubt; it is one of great interest from an anatomical and pathological stand-point. I am convinced that cases of this nature occur far more frequently than we imagine. The question of the evacuation of fluids from the frontal sinus into the antrum has been little considered. Such conditions do exist. A fine probe may be carried into the antrum upward into the frontal sinus. This was accomplished after removing the greater portion of the upper part of the antrum. While Dr. Fillebrown did not state much about the treatment, I would say that the secret of success in empyema of the antrum is to keep it thoroughly clean, removing fluids, pus, and any foreign substance that may be present. In my experience I have found in a number of cases pieces of tooth-roots and other substances that have found their way into the opening made for the purpose of securing drainage. In a case I have in mind the surgeon, who treated the patient with flexible rubber tubing, lost a piece of the tubing, which passed into the antrum and remained there for several months, and was a constant source of irritation to the membrane. I removed it and had fairly good success with the case, which was a very difficult one to control.

The paper read by Dr. Barrett opens new thoughts in the field of prosthetic dentistry. A careful following of the rules laid down by him in adjusting dentures, although not done with a view of the anatomical relations, was most beautifully exhibited to us by Dr. Palmer. Those of you who will examine that plate will find that if it had been made of soft material, so the muscles could work into it, it would be exactly like Dr. Barrett told us about. Its firmness is, in a great measure, due to the relation of the muscles described by Dr. Barrett. I regard both papers of the very highest order, and we feel very glad, indeed, that our section has been able to present material of this kind to the profession.

*Dr. Abbott.*—I want to say a word in corroboration of the statement of the position taken by Dr. Fillebrown in this matter,—that there is an opening or a drainage from the frontal sinus into the antrum. I have for a long time believed this to be the case, but I have not had an opportunity of examining the antrum sufficiently to be sure of it. I remember one case where the surgeon who had it in charge took it away before I had a chance to thoroughly examine it, but we suspected that the trouble was in the frontal sinus. In cases of abscess of the antrum I believe that absolute cleanliness is what is needed, but it is the most difficult thing to do. There is where the trouble arises. I have devised a number of instruments for this purpose, and, as you know, I have the spray instrument, which I use. Where it is impossible to place the agent I wish, without using the pressure behind, I use the multiple spray, which throws it all around. I wind a mass of cotton on a bit of stick or wisp of broom, and use it for a plug. It has worked better than anything else. I have treated several cases where the tubes have been in the mouth, and have removed the tubes and used plugs instead. Most of them get well, however, by leaving the antrum entirely alone. I know of a case where there was a large opening into which I could put my finger very easily. It had been treated by a gentleman from the country. I pulled out the pieces of cotton that were there, and gave the patient a prescription of one-sixty-fourth carbolic acid, and told him to wash it out himself, and it healed in a few months' time.

*Dr. Holly-Smith.*—The peculiar interest which attaches to my mind in regard to this suggestion of Dr. Fillebrown, relative to the anatomical arrangement, makes me recall some cases which I had with a throat and nose specialist in the temporary conditions following grippe, where examination has proved the cavity to be full of fluid, where no opening of any character has been made, and where recovery has resulted soon after the original trouble subsided. The statement made as to the leakage of the frontal sinus into the antrum was verified to me by a careful examination and experience with these cases. In reference to the treatment of these suppurating conditions in the antrum, I think that as a class we are too timid. I believe in a bolder operation. It is a well-known principle in surgery that we must have drainage for these conditions, and if men will heroically enter these cavities, cut out the floor, open up every part, curette the surface and pack with iodoform gauze,—instead of having a patient six months on your hands, those conditions will clear up in a short time. It is not necessary to make a marked deformity, which cannot be relieved; but I believe in an heroic operation for their relief. I have seen these cases treated for six years without relief, and an operation of the character which I have described has afforded relief in a few weeks.

*Dr. Barrett.*—We all recognize the importance of the subject, and I believe it to be an indisputable fact that dentists are about the poorest anatomists of those who practise the healing art; and yet no class of men should have a more intimate knowledge of anatomy, especially of the muscles of expression, than dentists. The horrible caricatures that we see in many mouths in which dentures are inserted, shows the lack of anatomical knowledge. I think dentists should study anatomy carefully, not only that they may restore the expression from the stand-point of nature, but that their prosthetic work may be a practical success.

In reference to Dr. Fillebrown's paper, I would say that last year Dr. Cryer presented a considerable number of anatomical sections, which were to me a revelation. He showed in some of these, tracing the course of the canal through the infundibulum until it discharged directly into the antrum, that it could not discharge into the meatus of the nose, but into the antrum itself. That was comparatively new to me. I had suspected something of the kind, but thought that there was a breaking down which had allowed the discharge to enter the antrum. It leads so dangerously near to the opening into the antrum that it was plainly evident that in many cases it might directly discharge into it. Dr. Cryer proved it, and showed it upon the screen. He exhibited to some of us these preparations, and he demonstrated that one thing perfectly. From that time my practice has been in that direction to a large extent, and I have modified it in regard to antral troubles most materially. I have found this: I do not know what I may meet next week or next month, but during the past year I have found no necessity whatever for the insertion of any tube into the antrum. I believe it to be unpathological and unphilosophical. It is an irritant, and causes a breaking down of the tissue. If there be an eroded surface, or empyema, perfect drainage will be of great benefit. If there be granulations of the mucous membrane around it, I remove them in some way, but that there be any tube, I do not think necessary. I have had some remarkable cases. I am very glad to have a paper like this read. It is something that calls our attention to a condition that exists that we should know all about. If we will watch the conditions and become fully acquainted with the real study of the case, I think it will materially modify our practice in all of the antral diseases, and there is no necessity for the violent measures which we adopted in the past, especially the packing of the antrum with a foreign substance of any kind, or the introduction of a drainage-tube when it can be drained without.

*Dr. Head.*—I want to speak of the statement that has been made, that there is no class of practitioners that has so little knowledge of anatomy as dentists. I would state that there is one class that has even less knowledge, and that is the general practitioner of medicine.

Dr. Crouse requested the members of the Dental Protective Association to meet him to-morrow afternoon at three o'clock.

Dr. Ambler then read a short paper, of which an abstract follows.

This is a report of a case which I had in my practice,—a nodule on the apex of a root. A lady, aged about thirty, presented for treatment a chronic abscess, the right superior lateral incisor. Upon examination with the probe, it was found that the labial wall of bone at the apex of the root had been destroyed. With the intention of scraping the apex and breaking up the sac, a small right-angle scoop was introduced and passed freely around and over the apex. Upon withdrawing it, a small nodule of dentine or enamel, about the size of an ordinary pin-head was brought away. The abscess was treated with pyrozone four times, at intervals of three days, when the discharge ceased. Evidently the pulp had been dead for a long time, and the canal had been filled, as there were large mesial and distal fillings. The case was lost sight of for a year, and in the mean time she had this lateral, also the right central, which was badly carious, extracted, and is now wearing an artificial denture. The special interest attached to this case is the fact of finding a nodule at the apex of the root and especially of a single-rooted tooth. Enamel nodules are small excrescences consisting of enamel occasionally met with upon the roots of the teeth. They are generally found upon multiple-rooted teeth, situated a little below the neck, and often at the junction of the roots. On dissection, they are found to consist of a cone of dentine covered with rather thick layers of enamel, and often connected to the crown by enamel. Wedl says that these nodules are the result of localized continuations of the development of the enamel between the already developed basal portion of the roots, and are produced by the strip of enamel organ which has persisted longer than the rest.

A cut of one of these nodules has been shown on the apex of a molar root, and the statement made that they may be accounted for by a budding of the tissues concerned in the process of formation of the tooth.

Tomes shows a cut of a nodule situated on the neck of a single-rooted superior cuspid. As far as I have been able to examine the dental literature upon this subject and this case, I find there has never been a case reported or a cut made like the one I have here noted. For that reason I thought it was worthy of being reported.

*Dr. Morrison.*—On inquiry of the different sections, I found there was nothing to be presented to this body upon the subject of the planting of teeth, and I do not like to have that go by default. It has been my good fortune to report to this body from year to year on such matters, and I merely want to report progress, and that I continue to plant teeth right along and do it with as much confidence as ever, and with as much confidence in those teeth rendering good service for a reasonable time as any other dental operation that is performed. There are no new features that I wish to present at this time, but merely report progress. On the subject of another question that I think is a little new I have something to say. Instead of constructing an apparatus to regulate the whole arch or several teeth in the arch at one time, I merely direct the force upon one, using the hint of the physiological action that nature develops on one side at a time, sometimes one a little in advance of the other. I regulate in the same manner. I put my force against one side at a time. In interlocked laterals drive one side right forward where you want it in a week or ten days; then make a retaining band with a yoke on the two adjoining teeth and reverse the apparatus, and put it on the other side.

*Dr. Fillebrown.*—Within a few weeks I have had the privilege of speaking upon this subject before a dental society, and at their request, and partly at my suggestion, I wrote to Dr. Cryer, and he kindly loaned me all the slides that he exhibited before this Association last year. I took the text of his paper which he produced at the time, and I exhibited them and followed the text of the paper. All I have to say is that in that assortment of slides and in the text of his paper that I reproduced, he did not say anything in regard to the points which I have produced here, nor showed any slides at all like the ones I show you to-night. In regard to anatomy I just want to point the moral that Dr. Barrett has well remarked, that our dentists do not know enough about it, and that is a supreme reason why our dental schools should teach anatomy from the bottom to the top. At the Harvard School the dental students and the medical students are taught together, and also in some other schools. I speak of this school especially, because I know about it. During Dr. Holmes's incumbency of the chair of anatomy in the Harvard Dental School, there were only three or four men who reached a standard of one hundred in anatomy, and one of those is a dentist who is to-day practising. Our men are smart enough. Give them the opportunity, and they will come out accomplished in that respect.

Dr. Richards, of Knoxville, Tenn., presented a paper entitled "The Morphology of Dental Pulps," which was illustrated by lantern slides.

Adjournment.

(To be continued.)

